# Development of functional gastrointestinal disorder symptoms following laparoscopic cholecystectomy: a prospective cohort study

**DOI:** 10.3389/fmed.2023.1248465

**Published:** 2023-10-06

**Authors:** Ji Young Chang, Hye-Kyung Jung, Chang Mo Moon, Seong-Eun Kim, Ki-Nam Shim, Sung-Ae Jung, Seog Ki Min

**Affiliations:** ^1^Department of Health Promotion Medicine, Ewha Womans University Seoul Hostpial, Seoul, Republic of Korea; ^2^Department of Internal Medicine, College of Medicine, Ewha Womans University, Seoul, Republic of Korea; ^3^Department of Surgery, College of Medicine, Ewha Womans University, Seoul, Republic of Korea

**Keywords:** diarrhea, dyspepsia, chronic abdominal pain, cholecystectomy, post-cholecystectomy syndrome

## Abstract

**Background:**

The casual relationship between the role of cholecystectomy and functional gastrointestinal disorders (FGIDs) are a controversial clinical challenge. This study aimed to investigate: (1) the overlap of FGIDs before cholecystectomy and its long-term outcome after surgery in patients with symptomatic cholelithiasis, and (2) the incidence of new-onset FGIDs after cholecystectomy.

**Methods:**

Patients with symptomatic gallstone disease who underwent elective, laparoscopic cholecystectomy were prospectively enrolled. Healthy populations who underwent medical check-ups were selected as age- and sex-matched controls. Questionnaires regarding sociodemographic characteristics, gastrointestinal symptoms and a somatization symptom checklist (SSC) were completed at baseline and 12 months thereafter.

**Results:**

The prevalence of all FGID symptoms before cholecystectomy were significantly higher in the group of patients with symptomatic cholecystolithiasis compared to the control group. In cholecystectomy group, the preoperative FGID symptoms improved after surgery, except for chronic diarrhea. Compared to the controls, the new-onset FGIDs, including functional dyspepsia (14.8% vs. 6.9%; *p* = 0.040), functional diarrhea (6.6% vs. 0.2%; *p* < 0.001), and chronic abdominal pain (11.9% vs. 4.4%; *p* = 0.024), were more common at 1 year after cholecystectomy. Somatization was independent predictors of new-onset dyspepsia and abdominal pain, while newly occurring diarrhea was not realted to somatization.

**Conclusion:**

Overlap of FGIDs was common in patients with symptomatic cholelithiasis before surgery and at follow-up 1 year after cholecystectomy. Furthermore, new-onset FGIDs could be occurred after cholecystectomy. Therefore, a delicate diagnostic approaches and appropriate treatments about co-existent FGIDs should be given in patients with cholelithiasis before and after cholecystectomy.

## Introduction

Functional gastrointestinal disorders (FGIDs) are common in the general population and comprise a major portion of clinical practice from primary to tertiary care (1, 2). Over the last decade, the pathophysiological mechanisms of FGIDs have been identified as intricate interactions among gastrointestinal (GI) motor dysfunction (3), visceral hypersensitivity (4), infections (5), alteration of the gut microbiota (6), dysfunction of the brain-gut axis (1), psychosocial factors including early life events (7), abuse (8), and social behavioral learning (9). In addition, the role of bile acids in the evolution of GI symptoms compatible with FGIDs has recently been reported (10). Bile acids are essential for digestion and the absorption of lipids and vitamins (11). They exert a laxative effect in the colon by secreting water and mucus, increasing motility and resulting in a metabolic effect and a cell turnover function (10). The slowing of gastric emptying and small bowel transit results in functional dyspepsia *via* G protein-coupled bile acid receptor activation (12). The negative feedback loop involving hepatic bile acid production has been identified as a cause of irritable bowel syndrome (IBS) symptoms or diarrhea in patients with intact intestines (13). Several studies have reported the relationship between bile acid dyshomeostasis and IBS (14). Especially, patients with diarrhea-predominant IBS are known to have elevated concentrations of fecal primary bile acids (15–17) which is associated with diarrhea and visceral pain. Additionally, recent conducted rodent study suggested that bile acid activated nociceptors and induce visceral hypersensitivity by upregulating Nerve Growth Factor, a mediator of pain generation and maintenance (18).

While iatrogenic manipulation of bile flow in humans is limited, cholecystectomy, a treatment choice for symptomatic gallstone disease is a model that could clearly reflect changes in bile circulation. Indeed, a significant proportion of patient experiences altered bowel symptoms after cholecystectomy, described as post-cholecystectomy syndrome which may be due to bile circulation change (19, 20). These symptoms include biliary pain or atypical GI symptoms similar with FGIDs. Moreover, FGIDs that often occur as a consequence of inflammation have recently gained more attention as a significant factor in their pathogenesis (21).

Therefore, we hypothesized that changes in bile circulation or inflammatory process relate to cholecystectomy in patients with symptomatic cholelithiasis would affect FGID symptoms. We conducted this study to investigate (1) the overlap of FGIDs before cholecystectomy and its long-term outcome after surgery in patients with symptomatic cholelithiasis, and (2) the incidence of new-onset FGIDs after cholecystectomy.

## Materials and methods

### Study participants

Patients who underwent elective laparoscopic cholecystectomy were recruited prospectively from May 2015 to December 2018. Patients aged 20–70 years who underwent elective laparoscopic cholecystectomy for symptomatic gall stone diseases were included. Age- and sex-matched patients who were evaluated for medical checks-ups at the health promotion center were randomly selected to be included in the control group. In the cholecystectomy group, patients who underwent emergency or open cholecystectomy, had postoperative complications, and had endoscopic procedures including endoscopic sphincterectomy or endoscopic balloon dilatation within 1 year and had biliary colic before cholecystectomy were excluded. And in the both group, patients with a history of major abdominal surgery, pelvic surgery or gynecologic surgery and chronic comorbidities that significantly affected the patient’s general condition (such as malignant disease, inflammatory bowel disease, pancreatic disease, chronic renal failure, liver cirrhosis, or chronic obstructive pulmonary disease) were also excluded from the study.

All patients provided written informed consent for their participation in this study. This study was approved by the Institutional Review Board of Ewha medical center (IRB number: ECT201-12).

### Questionnaires

All patients completed questionnaires at baseline (phase 1) and 12 months thereafter (phase 2). One day before cholecystectomy, study coordinator who was blinded to the study aim conducted the first phase questionnaires following screen of each patient’s appropriateness for study inclusion by another study coordinator. For the control group, first phase questionnaires were surveyed on check-up day.

During phase 1, the questionnaire gathered information regarding the patients’ sociodemographic information, weight and height, medical history, GI symptoms, and somatic symptom checklist (SSC). Structured questionnaire regarding GI symptoms were used to diagnose FGIDs, including functional dyspepsia, functional constipation, functional diarrhea, and IBS (based on the Rome III criteria). Chronic abdominal pain was defined as abdominal pain occurring at least 3 days per month during the past 3 months with the onset at least 6 months prior that was not diagnosed as IBS.

The incidence of disease was defined as newly developed symptoms within 12 months after baseline, and the persistence was defined as the cases which existed in both phases (22).

The SSC, a valid measure of psychosomatic complaints, (23) consists of 17 questions regarding non-GI symptoms and illnesses, including headaches, backaches, and insomnia. The intensity and frequency of each symptom or illness during the past year were scored from 0 to 4 (0 = not a problem; 4 = extremely bothersome/occurs daily). The final index score for somatic symptoms was calculated as the mean of the product of each item’s intensity and frequency.

Patients who underwent cholecystectomy also answered questions regarding coping strategies for stressful events. The coping strategies were classified as active cognitive, active behavioral, and avoidance-oriented strategies (24). Each statement regarding the frequency of each strategy was scored from 0 (not at all) to 3 (fairly often), and the average score was determined. The average score was divided by the sum of averages derived from the three strategies and expressed as a percentage to determine the relative score. High relative scores were used to identify each patient’s main coping strategy.

The phase 2 questionnaires only regarding GI symptoms were completed by each patient *via* a telephone interview 12 months after the first survey. English and Korean version of complete questionnaires in the Supplementary Data.

### Surgery protocol

The conventional, three-port, laparoscopic cholecystectomy technique was used with the patient in the supine position. After the abdomen was fully inflated with CO_2_, the neck of the gallbladder was retracted. The common bile duct was identified, and the cystic duct and cystic artery were resected. The gallbladder was detached from the liver bed with laparoscopic hook-bovie. The detached gallbladder was extracted through an umbilical incision and placed in a laparoscopic bag.

### Statistical analysis

All statistical analyses were conducted using SPSS, version 22.0 (SPSS Inc., Chicago, Illinois, United States). Continuous variables are presented as mean and standard deviation, and categorical variables are presented as frequency and percentage. The baseline clinical characteristics, and prevalence, incidence, and persistence of FGID symptoms were compared between the two groups using the *t* test or Mann–Whitney *U* test for continuous variables and the chi-square or the Fisher’s exact test for categorical variables. Univariate and multivariate logistic regression analyses were performed to identify independent predictors of new-onset FGIDs after cholecystectomy. Statistical significance was set at a *p*-value of <0.05.

## Results

### Baseline clinical characteristics of study participants

**Figure 1:**
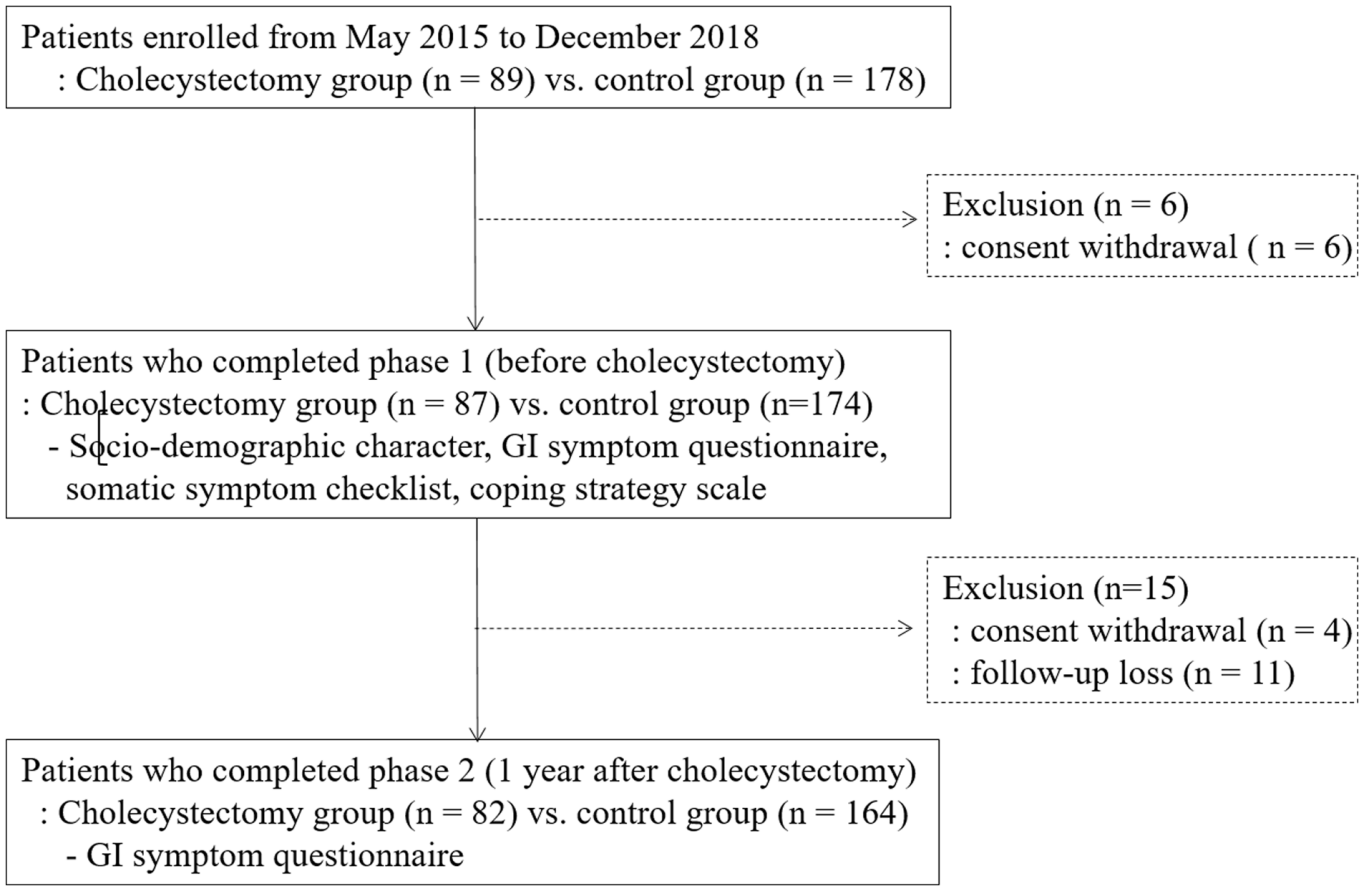
Study flow. GI, gastrointestinal.

A total of 261 patients (87 patients in the cholecystectomy group and 174 patients in the control group) completed the questionnaires during phase 1. The mean BMI (24.1 ± 3.9 vs. 23.2 ± 3.0; *p* = 0.03) and SSC score (1.2 ± 1.4 vs. 0.7 ± 1.0, *p* < 0.001) were significantly higher in the cholecystectomy group than in the controls (Table 1). Patients in the cholecystectomy group were more likely to have diabetes mellitus than those in the control group (8.4% vs. 4.1%, *p* = 0.04). The flow diagram of this study is shown in Figure 1.

**Table 1:** Baseline clinical characteristics of study participants.

Total (*n* = 261)	Cholecystectomy (*n* = 87)	Controls (*n* = 174)	*p* value
Age, mean ± SD	45.08 ± 11.80	46.04 ± 9.49	0.40
Sex (male), *n* (%)	53 (44.5)	290 (44.5)	0 > 0.99
BMI, mean ± SD (kg/m^2^)	24.08 ± 3.85	23.22 ± 3.02	0.03
Diabetes mellitus, n (%)	10 (8.4)	27 (4.1)	0.04
SSC score, mean ± SD	1.21 ± 1.35	0.72 ± 0.98	< 0.001

SD, standard deviation; BMI, body mass index; SSC, somatic symptom checklist.

### Prevalence of FGID symptoms during phase 1

**Figure 2:**
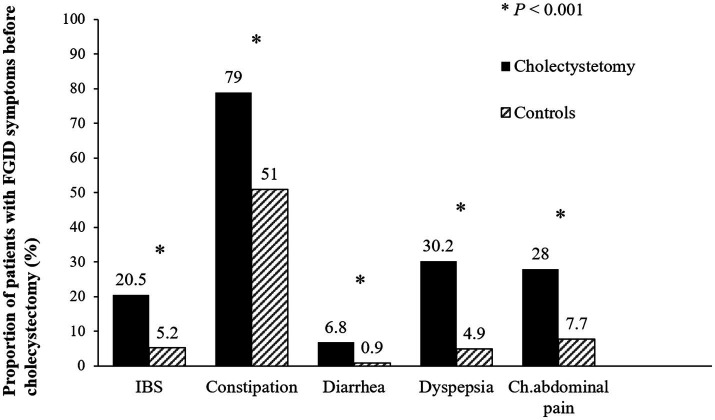
Prevalence of functional gastrointestinal disorders before surgery. The prevalence of irritable bowel syndrome, functional constipation, functional diarrhea, functional dyspepsia, and chronic abdominal pain were significantly higher in the cholecystectomy group than in the control group (all *p* < 0.001). FGID, functional gastrointestinal disorder; IBS, irritable bowel syndrome; Ch. abdominal pain, chronic abdominal pain.

The overall prevalence of FGID symptoms before surgery was significantly higher in the symptomatic cholelithiasis group than in the control group (82.9% vs. 59.1%, *p* < 0.001). IBS, functional constipation, functional diarrhea, functional dyspepsia and chronic abdominal pain were significantly more common in the cholecystectomy group than in the control group (all *p* < 0.001) (Figure 2).

### Prevalence and persistence of FGID symptoms during phase 2

**Figure 3:**
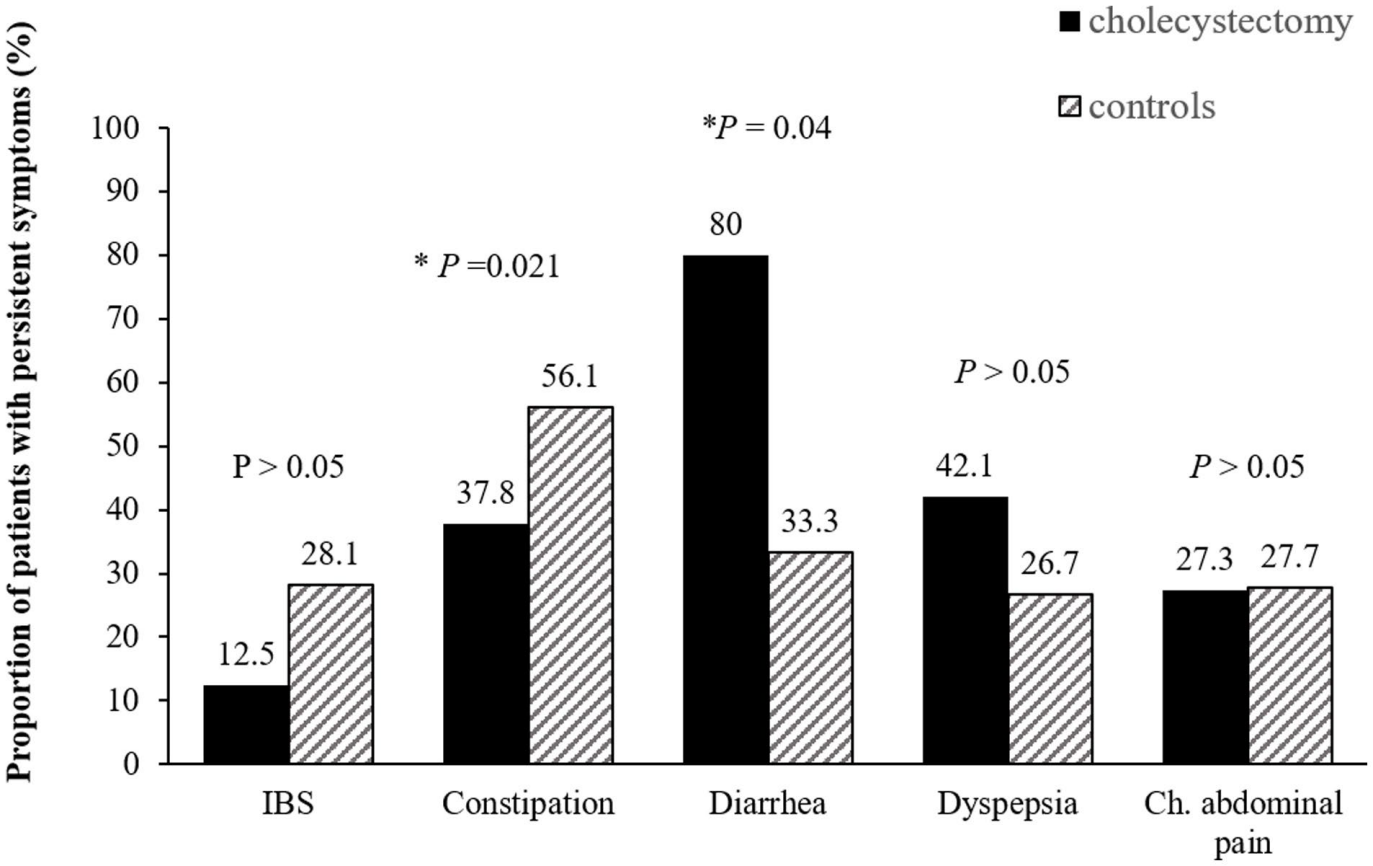
Persistence of functional gastrointestinal disorders after cholecystectomy. Compared to the control group, chronic constipation decreased after while chronic diarrhea significantly persisted. IBS, irritable bowel syndrome; Ch. abdominal pain, chronic abdominal pain.

A total of 82 patients in the cholecystectomy group and 164 in the control group completed the questionnaires during phase 2. After surgery, the overall prevalence showed a decreasing trend, with any overlap of FGIDs decreasing from 82.9% preoperatively to 51.2% postoperatively (Supplementary Figure S1). Compared to the control group, chronic constipation decreased after cholecystectomy (37.8% vs. 56.1%, *p* = 0.02) while chronic diarrhea significantly persisted (80.0% vs. 33.3%, *p =* 0.04 by Mann- Whitney *U* test). However, there was no significant differences in dyspepsia, IBS and chronic abdominal pain between cholecystectomy group and the control group after surgery (Figure 3).

### New-onset FGID symptoms during phase 2

**Figure 4:**
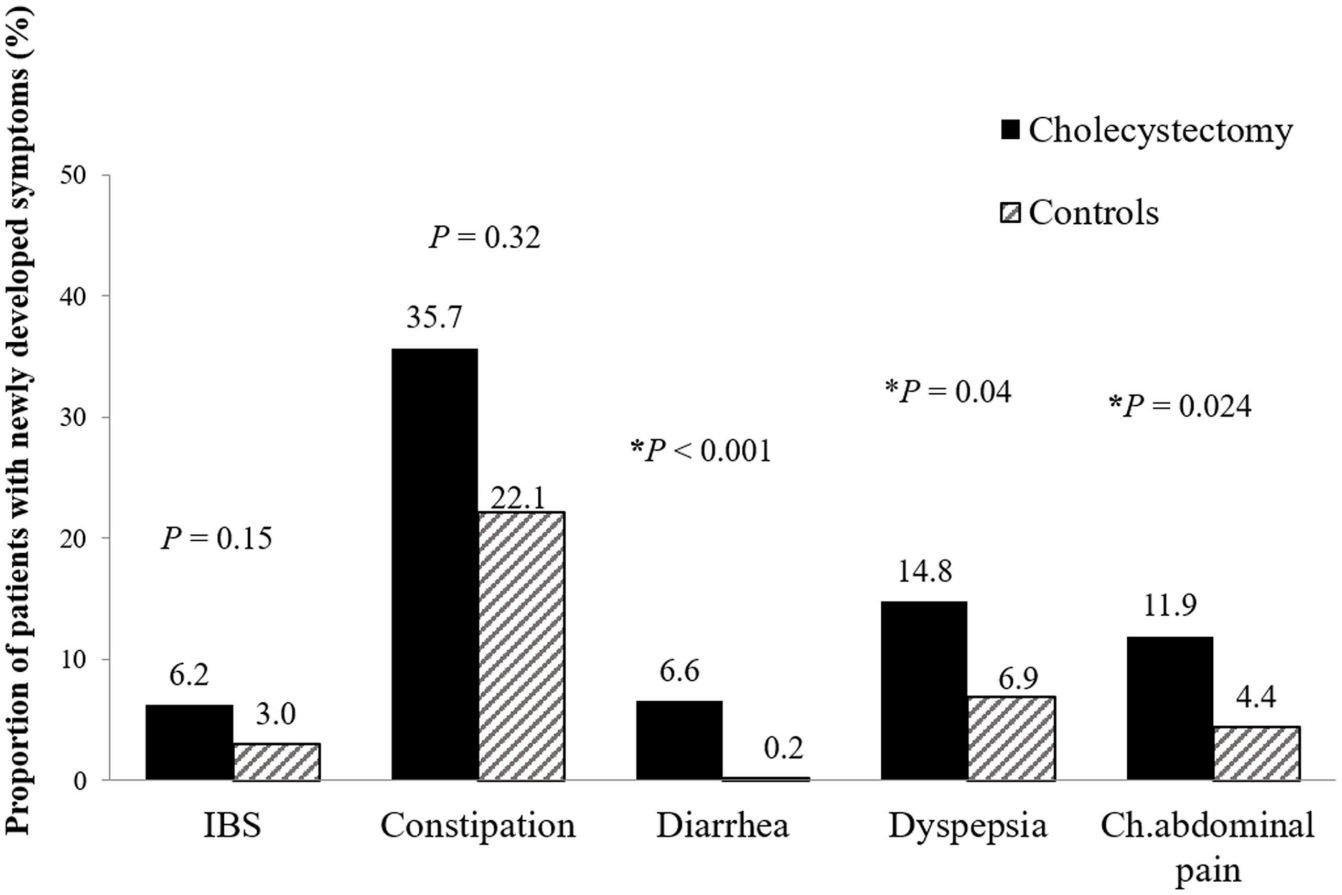
New-onset functional gastrointestinal disorders 1 year after cholecystectomy. Patients who underwent cholecystectomy had higher prevalence of new-onset functional diarrhea (6.6% vs. 0.2%; p < 0.001), dyspepsia (14.8% vs. 6.9%; *p* = 0.040), and chronic abdominal pain (11.9% vs. 4.4%; *p* = 0.024) than control patients. IBS, irritable bowel syndrome; Ch. abdominal pain, chronic abdominal pain.

One year after cholecystectomy, there was a significantly higher incidence of new-onset diarrhea (6.6% vs. 0.2%; *p* < 0.001), functional dyspepsia (14.8% vs. 6.9%; *p* = 0.04), and chronic abdominal pain (11.9% vs. 4.4%; *p* = 0.02) in the surgery group compared to the control group (Figure 4). However, new-onset IBS (6.2 vs. 3.0%, *p* = 0.21) and chronic constipation (35.7% vs. 22.1%, *p* = 0.18) were not significantly different between the groups.

### Predictors of new-onset FGID symptoms after cholecystectomy

To assess the effects of cholecystectomy on the development of individual symptoms of newly occurring FGIDs, we analyzed the variables affecting newly occurring FGIDs in both the surgery group and the control group as a whole (Table 2). Cholecystectomy [odds ratio (OR) = 2.32; 95% confidence interval (CI): 1.07–5.04], higher SSC score (OR = 1.89; 95% CI: 1.48–2.40) and male sex (OR = 0.50; 95% CI: 0.23–0.94) were identified as predictors for developing functional dyspepsia in univariate analysis (Supplementary Table S1). After adjusting multiple variables, including sex, age, cholecystectomy state, and SSC score using logistic regression analyses, cholecystectomy (OR = 2.29; 95% CI: 1.02–5.15) and high SSC (OR = 1.82; 95% CI: 1.43–2.31) were identified as independent predictors of new-onset functional dyspepsia. Cholecystectomy (OR = 2.62; 95% CI: 1.05–6.53) and high SCC scores (OR = 1.94; 95% CI: 1.41–2.68) were associated with the incidence of chronic abdominal pain. Only cholecystectomy (OR = 40.04; 95% CI: 4.50–356.26) was predictors of new-onset functional diarrhea. Univariate analysis results for new-onset chronic abdominal pain and functional diarrhea are shown in Supplementary Tables S2, S3, respectively.

**Table 2:** Multivariate analysis for predictors of new-onset functional gastrointestinal disorders after cholecystectomy.

	Functional dyspepsia			Functional diarrhea			Chronic abdominal pain		
	OR	95% CI	*p* value	OR	95% CI	*p* value	OR	95% CI	*p* value
Male	0.64	0.33–1.25	0.19	1.44	0.26–7.96	0.68	1.175	0.55–2.2	0.68
Age (years)									
≤ 30	1.45	0.49–4.35	0.50	–	–	0.99	0.662	0.08–5.57	0.70
31–50	1.06	0.54–2.07	0.87	–	–	0.99	1.744	0.73–4.15	0.21
≥ 51	1	–	1	1	–	1	1	–	1
Cholecystectomy	2.29	1.02–5.15	0.04	40.04	4.50–356.26	< 0.01	2.62	1.05–6.53	0.04
SSC score	1.82	1.43–2.31	< 0.01	1.31	0.77–2.24	0.32	1.94	1.41–2.68	< 0.01

OR, odd ration; CI, confidence interval; SSC, somatic symptom checklist.

### Influence of coping strategy on the changes of FGIDs after cholecystectomy

During phase 1, patients with FGID symptoms had lower active cognitive and active behavioral coping strategy scores and higher avoidance-oriented coping strategy scores than patients without FGID symptoms, although these differences were not statistically significant. During phase 2, the coping strategy scores were not different between patients with new-onset FGID symptoms and those without symptoms. However, patients with resolved FGID symptoms during phase 2 had significantly higher active cognitive coping strategy scores (42.7 ± 6.8 vs. 38.7 ± 7.1, *p* = 0.04) and lower avoidance-oriented coping strategy scores (20.7 ± 9.3 vs. 27.1 ± 9.4, *p* = 0.02) than patients with persistent FGID symptoms (Table 3).

**Table 3:** Impact of coping strategies on occurrence of functional gastrointestinal disorders after cholecystectomy.

	Active cognitive	Active behavioral	Avoidance-oriented
All patients (119)	40.5 ± 7.4	36.3 ± 6.8	23.2 ± 10.5
Baseline FGIDs			
No (18)	41.6 ± 9.4	39.0 ± 7.1	19.4 ± 12.0
Yes (101)	40.3 ± 7.0	35.8 ± 6.7	23.9 ± 10.1
*p* value	0.49	0.06	0.09
Incidence of FGIDs			
No (5)	43.7 ± 6.6	35.4 ± 9.0	20.9 ± 12.0
Yes (7)	41.3 ± 8.5	39.4 ± 6.9	19.3 ± 11.1
P value	0.60	0.40	0.82
Persistence of FGIDs			
No (21)	42.7 ± 6.8	36.6 ± 5.0	20.7 ± 9.3
Yes (32)	38.7 ± 7.1	34.3 ± 6.0	27.1 ± 9.4
*p* value	0.04	0.14	0.01

FGIDs, functional gastrointestinal disorders.

## Discussion

This study is the first prospective study that investigates changes in FGID symptoms after cholecystectomy in patients with symptomatic cholelitiasis and compares these changes to the healthy population. Before cholecystectomy, this group had a significantly higher prevalence of chronic symptom-compatible FGIDs, such as functional dyspepsia, functional constipation, diarrhea, and IBS, at 82.9% compared to the control group. While most symptoms improved after surgery, 51.2% continued to experience persistent FGIDs and new-onset FGIDs. Especially, new-onset dyspepsia and chronic abdominal pain occurred more frequently compared to the control group, and these symptoms were associated with somatization.

A previous study evaluated changes in dyspeptic (gas/flatulence, heartburn, belching) or biliary (nausea, food intolerance, vomiting, tenderness to touch) symptoms after cholecystectomy and reported that all symptoms decreased after surgery, although dyspeptic symptoms that were categorized as functional symptoms were more likely to persist or develop than biliary symptoms (25). However, the previous study did not include a control group or provide validated definitions. In the current study, the baseline prevalence of all FGID symptoms was significantly higher in the cholecystectomy group than in the control group. Patients with symptomatic gall stone disease may have precipitating factors leading to FGIDs. BMI and the prevalence of diabetes mellitus were significantly different between the two groups at baseline in this study, suggesting that these factors may be related to symptomatic gall stone disease. Unlikely the diagnosis of FGIDs in subjects without cholelithaisis, the diagnosis of overlapping FGIDs in symptomatic cholelithiasis requires investigation in order to exclude the active inflammation. Thus, in this study, only patients who underwent elective surgery were included, excluding those who underwent emergency surgery for active cholecystitis or experienced postoperative complications. A subset of FGIDs patients has a history of suggestive of post-infectious dyspepsia or post-infectious IBS (26, 27). In symptomatic cholelithiasis, acute or chronic low grade inflammatory changes in gall bladder might be attributable to be a dysfunction in visceral hypersensitivity. In addition to the traditional cholelithiasis-focused clinical history, particularly in the absence of active inflammation and other structural pathology, screening questions for positive diagnostic features of FGIDs are important before cholecystectomy. Acute or chronic biliary pain due to cholelithiasis may trigger FGIDs.

The most obvious change in GI symptoms after cholecystectomy is the change in bowel habits. Most FGID symptoms decreased in patients who underwent cholecystectomy; however, the prevalence of functional diarrhea significantly increased and new-onset functional diarrhea was more prevalent in the cholecystectomy group than in the control group. Post-cholecystectomy diarrhea occurred in 6.6% of patients in the current study, which is consistent with previously reported rates (2–8%) (28, 29). In a recent Taiwanese study, 25.2% of patients developed diarrhea 1 week after cholecystectomy, although this number decreased to 5.7% at 3 months postoperatively. No variables were identified as predictors of diarrhea at 3 months postoperatively; however, a high-fat diet and preoperative diarrhea were associated with the presence of diarrhea at 1 week postoperatively (30). After cholecystectomy, the elimination of reservoir function of gallbladder and bile acid malabsorption results in increased bowel motility and secretion. Considering that fecal bile acids are increased in up to 25% patients with functional diarrhea (31), bile circulation change after cholecystectomy seems to play a role in new-onset of diarrhea and decreased chronic constipation.

Dyspepsia and chronic abdominal pain developed frequently in patients who underwent cholecystectomy. Although several studies have evaluated the association between dyspeptic symptoms, gastric emptying, and cholecystectomy, the relationship and pathophysiology remain unclear. Gall stones have been reported to be associated with gastric motor dysfunction, and cholecystectomy may improve motility (32, 33). However, another study reported the improvement of delayed gastric emptying and gallstones after cholecystectomy, although no correlation between dyspeptic symptoms and delayed gastric motility was noted (34).

Several previous studies have reported the importance of psychological factors in the development of new-onset FGIDs. Sperber et al. (35), reported that the development of IBS was not significantly different between women who underwent gynecological surgery and control patients who did nog undergo surgery; nevertheless, the incidence of new-onset abdominal pain was significantly higher among patients who had undergone surgery. Psychological factors such as the anticipation of a difficult recovery from the operation, a low sense of coherence, perceived severity of the illness, and personal control were predictors of the development of new-onset abdominal pain, whereas sociodemographic and surgery-related factors were not predictive. These results highlight the importance of the brain-gut interaction in the pathophysiology of functional abdominal pain. Somatization disorders are common in patients with FGIDs (36), and often precedes or exacerbate FGID symptoms (37). Several studies have reported that the presence of IBS is associated with poor surgical outcomes after cholecystectomy or hysterectomy (38). In the current study, cholecystectomy status and high somatization score were associated with new-onset FGID symptoms. Female patients were more likely to have new-onset FGID symptoms, although this association was not observed in multivariate analyses. These results are compatible with those of a previous, longitudinal, population-based study (39).

This study has several strengths. First, the participants were enrolled in a prospective manner using validated symptom questionnaires. The prospective study design allows for systemic evaluation of the changes in major FGID symptoms after cholecystectomy using validated questionnaires and the Rome III criteria. Previous studies regarding post-cholecystectomy syndrome were retrospective or did not include a control group (40). Second, patients who underwent emergency surgery for acute cholecystitis or biliary obstruction and those who underwent open cholecystectomy were excluded from this study to avoid the effects of surgical stress. Lastly, this study included a relatively long follow-up period of 1 year.

In addition, this study is not without limitations. First, as the study was conducted at a tertiary referral center, selection bias cannot be ruled out. Patients with FGID who are referred to specialists may exhibit more severe manifestations of the diseases or may be more likely to seek surgical cure for their symptoms. Therefore, the high prevalence of abdominal surgeries in these patients may reflect differences in their natural histories after cholecystectomy. Second, physiological evaluation of bile flow changes to identify underlying pathogeneses was not conducted in this study. Physiological assessments of how changes in bile flow affect the GI tract should be included in future studies.

## Conclusion

Overlap of FGIDs was common in patients with symptomatic cholelithiasis before surgery compared to healthy controls. And at follow-up one year after cholecystectomy, significant patients experienced sustained FGIDs symptoms and, furthermore, new-onset functional diarrhea, functional dyspepsia, and chronic abdominal pain could be occurred after cholecystectomy. Therefore, a delicate diagnostic approaches and appropriate treatments about co-existent FGIDs using dietary, pharmacological, or psychological approaches should be given in the patients with cholelithiasis before and after cholecystectomy.

## Data availability statement

The original contributions presented in the study are included in the article/Supplementary material, further inquiries can be directed to the corresponding author.

## Ethics statement

This study was approved by the Institutional Review Board of Ewha medical center (IRB number: ECT201-12). The studies were conducted in accordance with the local legislation and institutional requirements. The participants provided their written informed consent to participate in this study.

## Author contributions

H-KJ and JC were responsible for conceiving the idea of the study and collecting the data, study design, data analysis, and manuscript writing. S-EK, CM, K-NS, and S-AJ reviewed this manuscript. SM was responsible for patient recruitment and questionnaire administration. All authors contributed to the article and approved the submitted version.

## Abbreviations

FGIDs: functional gastrointestinal disorders
SSC: somatization symptom checklist
GI: gastrointestinal
IBS: irritable bowel syndrome
BMI: body mass index
