# Supplementary material for: Development of functional gastrointestinal disorder symptoms following laparoscopic cholecystectomy: a prospective cohort study
Source: Front Med (Lausanne). 2023 Oct 6;10:1248465. doi: 10.3389/fmed.2023.1248465 (PMC10587431; doi:10.3389/fmed.2023.1248465)
Supplement: Supplementary file 1 [file Data_Sheet_1.docx]

Supplementary Material

# Supplementary Data.

**1.1 Gastrointestinal Symptom Questionnaire**

**Gastrointestinal Symptom Questionnaire (Phase 1)**

This gastrointestinal questionnaire is designed to more objectively investigate patient symptoms. It will be of great help in diagnosis and treatment.

Please complete the survey to the end.

Date: _______________Y ___________ M ____________ D

Name: _______________________________

Hospital ID_____________________________

Sex: Man ______Woman ______

1. During the past 3 months, have you ever felt discomfort or pain in your stomach or anywhere in your abdomen? (Please exclude chest pain or abdominal pain during menstruation).

0 □ No Please go to page 2, number 12.

1 □ Yes Please continue taking the survey below.

**Pain or discomfort in the stomach or abdomen is often difficult to express or occurs in alternation of two or more symptoms. Please think about the most common or most severe abdominal pain, or discomfort you experience and answer the questions below.**

2. Have you experienced this type of abdominal pain or discomfort more than 6 times in the past year?

0 □ No 1 □ Yes

3. Have you had above mentioned abdominal pain or discomfort for 6 months?

0 □ No 1 □ Yes

4. How often have you experienced this abdominal pain or discomfort in the last 3 months? (Please select only one answer)

0 □ none

1 □ <1 day/month

2 □ 1 day/month

3 □ 2-3 days/momth

4 □ 1 day/week

5 □ 2-6 days/week

6 □ daily

5. Where do you usually feel pain or discomfort in your abdomen? Please check the photo below.


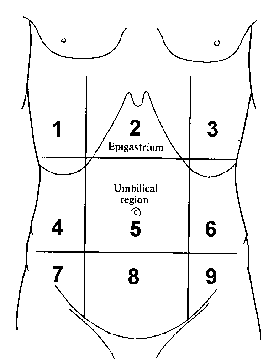
□ 1

□ 2

□ 3

□ 4

□ 5

□ 6

□ 7

□ 8

□ 9

6. How severe was the abdominal pain or discomfort described above?

(Please choose only one most suitable)

1 □ It is so mild that it is mostly unrecognized.

2 □ It is a mild symptom and cannot be felt unless you consciously think about it.

3 □ It exists to some extent but does not affect daily life.

4 □ It is severe enough to affect daily life.

5 □ It is so severe that daily life is difficult.

***Next, we would like to ask you about your bowel habits over the past three months. “Sometimes” means about 25% of bowel movements: “Often” means 50% of bowel movements: “Most of the time” means about 75% of bowel movements: “Always” means symptoms occur in 100% of bowel movements.***

7. In the past 3 months, has your abdominal pain or discomfort gotten better after having a bowel movement? (Choose the one that suits you best)

0 □ None or rare

1 □ Sometimes

2 □ Often

3 □ Mostly

4 □ Always

8. In the past 3 months, have you had a bowel movement more often when you have an abdominal pain or discomfort?

0 □ None or rare

1 □ Sometimes

2 □ Often

3 □ Mostly

4 □ Always

9. In the past 3 months, have you had abdominal pain or discomfort and had your bowel movements become more infrequent?

0 □ None or rare

1 □ Sometimes

2 □ Often

3 □ Mostly

4 □ Always

10. In the past 3 months, have your stools become soft or loose when you had abdominal pain or discomfort?

0 □ None or rare

1 □ Sometimes

2 □ Often

3 □ Mostly

4 □ Always

11. In the past 3 months, have your stools ever become harder when you have abdominal pain or discomfort?

0 □ None or rare

1 □ Sometimes

2 □ Often

3 □ Mostly

4 □ Always

12. How many times a week do you have a bowel movement? (Choose the one that suits you best)

1 □ ≤ 1

2 □ 2

3 □ 3-4

4 □ 5-8

5 □ 9-12

6 □ 13-16

7 □ 17-21

8 □ 22-26

9 □ ≥ 27

13. What does your final stool look like? (Choose the one that suits you best)


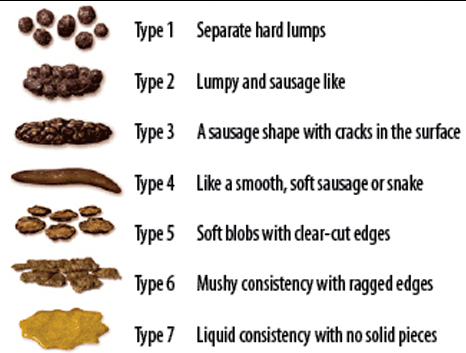


1 □ Type 1

2 □ Type 2

3 □ Type 3

4 □ Type 4

5 □ Type 5

6 □ Type 6

7 □ Type 7

14. In the past 3 months, have you ever had blood in your stool or toilet after a bowel movement? (Select all that apply)

0 □ No

1 □ Yes – bright red blood

2 □ Yes – black or very dark brown

15. In the past 3 months, have you had hard or lumpy stools? (Choose the one that suits you best)

0 □ None or rare

1 □ Sometimes

2 □ Often

3 □ Mostly

4 □ Always

16. In the past 3 months, have you ever strained excessively when having a bowel movement?

(Choose the one that suits you best)

0 □ None or rare

1 □ Sometimes

2 □ Often

3 □ Mostly

4 □ Always

17. In the past 3 months, have you ever had the feeling that feces remain in your anus after a bowel movement? (Choose the one that suits you best)

0 □ None or rare

1 □ Sometimes

2 □ Often

3 □ Mostly

4 □ Always

18. In the past 3 months, have you ever had trouble passing stool or felt like stool is stuck inside of your rectum?

0 □ None or rare

1 □ Sometimes

2 □ Often

3 □ Mostly

4 □ Always

19. In the last 3 months, have you ever pressed on the pelvic floor area or used your fingers to manually remove the stool? (Choose the one that suits you best)

0 □ None or rare

1 □ Sometimes

2 □ Often

3 □ Mostly

4 □ Always

20. If you have symptoms related to constipation that correspond to questions 15-19 above, did the symptoms begin 6 months ago?

0 □ No 1 □ Yes

21. Are you taking or using any of the following treatments for constipation? (Select all that apply)

0 □ None

1 □ Brown rice or mixed grains

2 □ Dietary fiber

3 □ Laxatives (Magmil, Dulcolax, Araxyl, etc.)

4 □ Enema 5 □ Suppositories (glycerin, Dulcolax, etc.)

22. In the last 3 months, have you had loose stools or watery stool?

0 □ None or rare -> Go to question 23

1 □ Sometimes.

2 □ Often.

3 □ Mostly

4 □ Always

22A. Did the above symptoms start 6 months ago?

0 □ No 1 □ Yes

22B. Did the above symptoms occur after taking laxatives or other laxatives?

0 □ No 1 □ Yes

23. In the last 3 months, have you had fecal incontinence (uncontrolled excretion of feces)?

0 □ None -> Go to question 24

1 □ Less than once a month

2 □ 1 day per month

3 □ 2-3 days per month

4 □ One day a week

5 □ 2-6 days a week

6 □ Every day

23A. When you had fecal incontinence, the amount was

1 □ stain underwear

2 □ less than usual, and need to change underwear

3 □ similar to usual, and need to change outerwear

23B. When you had fecal incontinence, the shape was

1 □ Only water or mucus

2 □ Only stool

3 □ Water, mucus, and stool

***Next, we would like to ask you about other gastrointestinal symptoms.***

24. In the last 3 months, have you ever had throbbing or burning pain upward from the epigastric area to the chest? (exclude pain due to angina pectoris or heart disease) (Choose the one that suits you best).

0 □ None -> Go to question 25

1 □ Less than once a month

2 □ 1 day per month

3 □ 2-3 days per month

4 □ 1 day a week

5 □ 2-6 days a week

6 □ Every day

24A. Did the above symptoms start 6 months ago?

0 □ No 1 □ Yes

24B. Have you ever had gastric juice come up into your mouth in the last 3 months?

0 □ No 1 □ Yes

25. In the last 3 months, have you ever felt full so quickly that you could only eat a small meal?

(Choose the one that suits you best)

0 □ None -> Go to question 25

1 □ Less than once a month

2 □ 1 day per month

3 □ 2-3 days per month

4 □ 1 day a week

5 □ 2-6 days a week

6 □ Every day

26. In the last 3 months, have you had a feeling that your upper abdomen is full (not going down) after finishing a normal meal? (Choose the one that suits you best)

0 □ None -> Go to question 25

1 □ Less than once a month

2 □ 1 day per month

3 □ 2-3 days per month

4 □ 1 day a week

5 □ 2-6 days a week

6 □ Every day

27. Did the symptoms in questions 25-26 above begin 6 months ago?

0 □ No 1 □ Yes

28. In the past 3 months, have you ever have the pain in upper abdomen [epigastric area]?

(Choose the one that suits you best)

0 □ None -> Go to question 29

1 □ Less than once a month

2 □ 1 day per month

3 □ 2-3 days per month

4 □ 1 day a week

5 □ 2-6 days a week

6 □ Every day

28A. How severe was the pain in your upper abdomen?

1 □ It is so mild that it is mostly unrecognized.

2 □ It is a mild symptom and cannot be felt unless you consciously think about it.

3 □ It exists to some extent but does not affect daily life.

4 □ It is severe enough to affect daily life.

5 □ It is so severe that daily life is difficult.

28B. Have you had these symptoms for more than 6 months?

0 □ No 1 □ Yes

28C. Does the pain get better after having a bowel movement?

0 □ No 1 □ Yes

28D. Do the symptoms be improve in a day?

0 □ No 1 □ Yes

28E. These symptoms

0 □ are not related to the eating.

1 □ get better after eating.

2 □ get worse after eatingt.

29. In the past 3 months, have you ever felt bloating? (Choose the one that suits you best)

0 □ None -> Go to question 30

1 □ Less than once a month

2 □ 1 day per month

3 □ 2-3 days per month

4 □ 1 day a week

5 □ 2-6 days a week 6 □ Every day

29A. When you have a bloating, do you actually feel like your stomach is swollen?

0 □ None or rare

1 □ Sometimes

2 □ Often

3 □ Mostly

4 □ Always

29B. Have you had these symptoms for more than 6 months?

0 □ No 1 □ Yes

30. In the past 3 months, have you ever felt nauseous? (Choose the one that suits you best)

0 □ None -> Go to question 31

1 □ Less than once a month

2 □ 1 day per month

3 □ 2-3 days per month

4 □ 1 day a week

5 □ 2-6 days a week

6 □ Every day

30A. Have you had these symptoms for more than 6 months?

0 □ No 1 □ Yes

31. In the past 3 months, have you actually vomited? (Choose the one that suits you best)

0 □ None -> Go to question 31

1 □ Less than once a month

2 □ 1 day per month

3 □ 2-3 days per month

4 □ 1 day a week

5 □ 2-6 days a week

6 □ Every day

32. In the past year, your weight

0 □ is not changed.

1 □ has increased less than 5kg.

2 □ has increased more than 5kg.

3 □ has decrease less than 5 kg.

4 □ has decrease more than 5 kg

33. Have you ever had abdominal surgery?

0 □ None.

1 □ Appendectomy.

2 □ Cholecystectomy.

3 □ Hysterectomy.

4 □ Other

34. During the past year, have you ever experienced urinary urgency?

0 □ None or rare

1 □ Sometimes

2 □ Often

3 □ Mostly

4 □ Always

35. Have you experienced urinary incontinence (the inability to hold urine until you go to the bathroom and the leaking of urine into your underwear) during the past year?

0 □ No 1 □ Yes

36. Have you ever been pregnant? (only include cases where the pregnancy lasted more than 6 months)? Please answer only if you are a woman.

0 □ No 1 □ Yes

If your pregnancy lasted more than 6 months, how many times did you give birth?

36A. Normal delivery (not stillbirth) ______________

36B. Cesarean section ______________

36C. Normal vaginal delivery ______________

37. What is your current marital status?

1 □ Single

2 □ Marriage

3 □ Divorce

4 □ Loss of spouse

5 □ Other ( )

38. What is your current job?

1 □ Retirement

2 □ Unable to get a job due to physical disability

3 □ Student or housewife

4 □ unemployed

5 □ Employment status

39. What is your highest level of education?

1 □ Uneducated

2 □ Graduation from elementary school

3 □ Graduated from middle school

4 □ Graduated from high school

5 □ Graduated from junior college

6 □ Graduated from a 4-year university

7 □ Graduate school or higher

40. How tall are you? (Please answer in centimeters) _______________

41. How much do you weigh? (Please answer in kilograms) _______________

42. Do you smoke regularly?

0 □ No

1 □ I used to smoke, but I quit now.

2 □ Yes

43. How much do you drink?

0 □ Never or less than once a month

1 □ 2-3 times a month

2 □ 1-2 times a week

3 □ More than 3 times a week

4 □ Drink every day.

44. Are you taking aspirin (including infant aspirin) or painkillers?

1 □ Never or less than 2-3 times a month

2 □ 1-2 times a week

3 □ 3-6 times a week

4 □ 7-10 times a week

5 □ More than 10 times a week

45. In the past 10 years, have you ever been told by a doctor that you have any of the following diseases?

45A. Any malignant disease 0 □ No 1 □ Yes (final diagnosis: )

45B. Diabetes mellitus 0 □ No 1 □ Yes

45C. Chronic renal disease 0 □ No 1 □ Yes

46. ​​How many times have you visited the doctor in the past year?

0 □ None.

1 □ 1-2 times

2 □ 3~5 times

3 □ 6~9 times

4 □ More than 10 times

46A. If you have ever visited a doctor, have you ever visited for digestive problems such as gastric or intestinal symptoms?

0 □ No 1 □ Yes

***Please check 1) how often and 2) how severe the following symptoms were during the past year (If not present, please check “none”).***

| **Frequency** | | | | |  | **Severity** | | | | |
| --- | --- | --- | --- | --- | --- | --- | --- | --- | --- | --- |
| None | More than once a month | More than once a week | several times a week | everyday |  | None | slightly uncomfortable | quite uncomfortable | Severe | Very severe |
|  |  |  |  |  | 1. Headache |  |  |  |  |  |
|  |  |  |  |  | 2. Back or lower back pain |  |  |  |  |  |
|  |  |  |  |  | 3. Asthma [wheezing] |  |  |  |  |  |
|  |  |  |  |  | 4. Dyspnea |  |  |  |  |  |
|  |  |  |  |  | 5. Insomnia [Sleep  difficult] |  |  |  |  |  |
|  |  |  |  |  | 6. Fatigue |  |  |  |  |  |
|  |  |  |  |  | 7. General stiffness |  |  |  |  |  |
|  |  |  |  |  | 8. Heart pounding |  |  |  |  |  |
|  |  |  |  |  | 9. Joint pain |  |  |  |  |  |
|  |  |  |  |  | 10. Depressed mood |  |  |  |  |  |
|  |  |  |  |  | 11. Ocular pain when looking at letters |  |  |  |  |  |
|  |  |  |  |  | 12. Dizziness |  |  |  |  |  |
|  |  |  |  |  | 13. General weakness |  |  |  |  |  |
|  |  |  |  |  | 14. Irritable or easily anxious |  |  |  |  |  |
|  |  |  |  |  | 15. Suddenly feels cold or hot |  |  |  |  |  |
|  |  |  |  |  | 16. High blood pressure |  |  |  |  |  |
|  |  |  |  |  | 17. Worried and scared |  |  |  |  |  |

***The following questions are designed to find out what you do when a difficult problem or difficult task arises. What has been the most difficult thing in the past three months? Think about what you did when that happened, and answer the following questions with numbers.***

0 (Not at all) 1 (A little) 2 (A bit) 3 (Very much)

1. I thought of several ways to solve the problem. ( )

2. I found out more about the situation. ( )

3. I talked about the problem with my spouse or relatives. ( )

4. I tried to look at the positive side. ( )

5. I prayed for help or strength. ( )

6. I talked to a friend about the problem. ( )

7. I talked to experts (doctors, lawyers). ( )

8. I busied myself with other things to get my mind off it. ( )

9. I made a plan for what to do and did it. ( )

10. When I was angry or depressed, I got irritated with others. ( )

11. I stored up my emotions in my heart. ( )

12. I ate more than usual. ( )

13. I thought carefully about what I did when something similar to this happened in the past. ( )

14. I thought about it step by step. ( )

15. I walked around the market or department store and looked around or bought things. ( )

16. I drank more alcohol than usual. ( )

17. Avoiding being around people. ( )

18. I refused to believe it happened. ( )

19. I smoked more cigarettes than usual. ( )

20. I slept more than usual. ( )

21. I put in more effort to accomplish what I need to do. ( )

22. I tried to express my feelings somehow. ( )

23. I thought of things that would make me feel better. ( )

24. I sought help from people with similar experiences. ( )

25. It was a difficult situation, but I tried to overcome it somehow. ( )

26. I promised myself I would be different next time. ( )

27. The problem was accepted as is. ( )

28. I took a sedative. ( )

29. I tried to relax by exercising harder. ( )

**Gastrointestinal Symptom Questionnaire (Phase 2)**

**Hospital ID ___________________________________**

**A1.** During the past 3 months, have you ever felt abdominal discomfort or pain?

0. No → [Go to A7] 1. Yes → [Go to A1-1]

A1-1. Have you had above mentioned abdominal pain or discomfort for 6 months?

0 □ No 1 □ Yes

A1-2. How often have you experienced this abdominal pain or discomfort?

1 □ <1 day/month

2 □ 1 day/month

3 □ 2-3 days/momth

4 □ 1 day/week

5 □ 2-6 days/week

6 □ daily

*** Regarding the abdominal pain or discomfort you have experienced in the past 3 months [A1], please refer to the following: Please choose one option that best describes your situation.**

|  | None or rare | Sometimes | Often | Mostly | Always |
| --- | --- | --- | --- | --- | --- |
| **A2.** Has your abdominal pain or discomfort gotten better after having a bowel movement? | 1 | 2 | 3 | 4 | 5 |
| **A3.** Have you had a bowel movement more often when you have an abdominal pain or discomfort? | 1 | 2 | 3 | 4 | 5 |
| **A4.** Have you had abdominal pain or discomfort and had your bowel movements become more infrequent? | 1 | 2 | 3 | 4 | 5 |
| **A5.** Have your stools become soft or loose when you had abdominal pain or discomfort? | 1 | 2 | 3 | 4 | 5 |
| **A6.** Have your stools ever become harder when you have abdominal pain or discomfort? | 1 | 2 | 3 | 4 | 5 |

* **Please choose one option that best describes your situation.**

|  | **none** | <1 day/ month | 1 day/ month | 2-3 days/ month | 1 day/ week | 2-6 days/ week | **daily** | |
| --- | --- | --- | --- | --- | --- | --- | --- | --- |
| **A7.** Have you ever had throbbing or burning pain in the epigastric area? | 0 | 1 | 2 | 3 | 4 | 5 | 6 |  |
| A7-1. Did the above symptoms start 6 months ago? | 0 □ No 1 □ Yes | | | | | | |  |
| **A8.** Have you ever felt full so quickly that you could only eat a small meal? | 0 | 1 | 2 | 3 | 4 | 5 | 6 |  |
| A8-1. Did the above symptoms start 6 months ago? | 0 □ No 1 □ Yes | | | | | | |  |
| **A9.** Have you had a feeling that your upper abdomen is full (not going down) after finishing a normal meal? | 0 | 1 | 2 | 3 | 4 | 5 | 6 |  |
| A9-1. Did the above symptoms start 6 months ago? | 0 □ No 1 □ Yes | | | | | | |  |
| **A10.** Have you ever had pain or burning sensation in the epigastric area? | 0 | 1 | 2 | 3 | 4 | 5 | 6 |  |
| A10-1. Did the above symptoms start 6 months ago?? 0 □ No 1 □ Yes | | | | | | | |  |
| A10-2. How severe was the symptoms described above?  1. Negligible 2. Mild 3. Moderate 4. Severe 5. Very severe | | | | | | | |  |

* **Please choose one option that best describes your situation.**

|  | | **none** | <1 day/ month | 1 day/ month | 2-3 days/ month | 1 day/ week | 2-6 days/ week | **daily** |
| --- | --- | --- | --- | --- | --- | --- | --- | --- |
| **A11.** | Have you ever had gastric juice come up into your mouth in the last 3 months? | 0 | 1 | 2 | 3 | 4 | 5 | 6 |
| **A12.** | In the past 3 months, have you ever felt nauseous? | 0 | 1 | 2 | 3 | 4 | 5 | 6 |
| **A13.** | In the past 3 months, have you actually vomited? | 0 | 1 | 2 | 3 | 4 | 5 | 6 |
| **A14.** | In the past 3 months, have you ever felt bloating or fullness? | 0 | 1 | 2 | 3 | 4 | 5 | 6 |

* **Please choose one option that best describes your situation.**

|  | **None or rare** | **Sometimes** | **Often** | **Mostly** | **Always** |
| --- | --- | --- | --- | --- | --- |
| **A24.** In the past 3 months, have you had hard or lumpy stools? | 1 | 2 | 3 | 4 | 5 |
| **A25.** In the past 3 months, have you ever strained excessively when having a bowel movement? | 1 | 2 | 3 | 4 | 5 |
| **A26.** In the past 3 months, have you ever had the feeling that feces remain in your anus after a bowel movement? | 1 | 2 | 3 | 4 | 5 |
| **A27.** In the past 3 months, have you ever had trouble passing stool or felt like stool is stuck inside of your rectum? | 1 | 2 | 3 | 4 | 5 |
| **A28.** In the last 3 months, have you ever pressed on the pelvic floor area or used your fingers to manually remove the stool? | 1 | 2 | 3 | 4 | 5 |
| **A29.** In the last 3 months, have you had loose stools or watery stool? | 1 | 2 | 3 | 4 | 5 |

**A30.** How many times a week do you have a bowel movement? ____________________________

1**.2** **Gastrointestinal Symptom Questionnaire in Korean**

**위장관 증상 설문지 (Phase 1)**

본 위장관 설문지는 환자분의 증상을 보다 객관적으로 알아보고자 하는 목적으로

시행하며 진단 및 치료에 큰 도움이 될 것입니다.

해당사항이 없는 증상도 대답하여 주시고 마지막까지 설문에 응해 주시기 바랍니다.

날짜: _______________년 ___________ 월 ____________ 일

이름: _______________________________

병원 번호:_____________________________

성별: ______ 남 ______ 여

**최근3개월간 위장관 불편감이나 통증이 있었는지에 관한 질문입니다..**

1. 최근 3개월 동안 위 혹은 배 어디든지에 불편감이나 복통이 있었습니까? *(가슴 통증이나 여성의 경우 생리기간에 일어나는 복통은 제외하여 주십시요)*

0 □ 아니요 2 페이지 12번으로 가세요

1 □ 예 아래 설문을 계속하여 주세요

**위나 배에 생기는 통증 혹은 불편감은 종종 표현하기 어렵거나 한가지 이상의 증상이 번갈아 나타납니다. 당신이 갖고 있는 위나 배의 통증 혹은 불편감 중에서 주로 일어나거나 가장 심한 증상에 대해 생각하시고 아래 질문에 답하여 주십시오.**

2. 지난 1년동안 이러한 복부 통증이나 불편감이 6번 이상 있었습니까?

0 □ 아니오

1 □ 예

3. 이러한 복부 통증이나 불편감이 6개월이상 되었습니까?

0 □ 아니오

1 □ 예

4. 최근 3개월동안, 얼마나 자주 이런

복부 통증이나 불편감이 있었습니까?

*(한가지 답만 선택하세요)*

5. 당신이은 주로 어느 부위에서 복부

통증이나 불편감을 느끼시는지 아래

그림에서 해당하는 곳을 모두 고르세요.


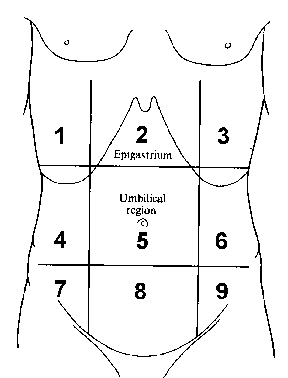


0 □ 없음

1 □ 1달에 한 번 미만

2 □ 1 달에 1일

3 □ 1달에 2-3 일

4 □ 1주일에 하루

5 □ 1주일에 2-6일

6 □ 매일

□ 1

□ 2

□ 3

□ 4

□ 5

□ 6

□ 7

□ 8

□ 9

6. 위의 복부 통증이나 불편감이 어느

정도로 심했습니까? *(가장 적합한*

*한가지만 고르세요)*

1 □ 아주 가벼워 대부분 잘 모른다.

2 □ 가벼운 정도로 생각하지 않으면 잘 모른다.

3 □ 어느 정도 있으나 일상생활에 영향을 주지 않는다.

4 □ 심해 일상생활에 영향을 준다.

5 □ 매우 심해 일상생활이 어렵다.

| **다음으로 최근 3개월간 귀하의 배변 습관에 관해 질문하고자 합니다.**  **“가끔 그렇다” 는 배변의 25% 정도: “자주 그렇다”는” 배변의50%: “대부분 그렇다” 는 배변의 75% 정도: “항상 그렇다”는 배변의 100% 에서 증상이 있는 경우입니다 .** |
| --- |

7. 최근 3개월 동안 배가 아프거나 불편한 증상이 대변을 보고 나면 좋아지나요? *(가장 적합한 한가지만 고르세요)*

8. 최근 3개월 동안 배가 아프거나 불편한 증상이 있을 때 대변을 더 자주 보나요?

0 □ 전혀 아니거나 거의 드물다.

1 □ 가끔 그렇다.

2 □ 자주 그렇다.

3 □ 대부분 그렇다.

4 □ 항상 그렇다.

0 □ 전혀 아니거나 거의 드물다.

1 □ 가끔 그렇다.

2 □ 자주 그렇다.

3 □ 대부분 그렇다.

4 □ 항상 그렇다.

9. 최근 3개월 동안 배가 아프거나 불편한 증상이 있을 때 대변을 더 드물게 보나요?

0 □ 전혀 아니거나 거의 드물다.

1 □ 가끔 그렇다.

2 □ 자주 그렇다.

3 □ 대부분 그렇다.

4 □ 항상 그렇다.

10. 최근 3개월 동안 배가 아프거나 불편할 때 대변이 무르거나 풀어지나요?

11. 최근 3개월 동안 배가 아프거나 불편할 때 대변이 더 단단하거나 굳어지나요?

0 □ 전혀 아니거나 거의 드물다.

1 □ 가끔 그렇다.

2 □ 자주 그렇다.

3 □ 대부분 그렇다.

4 □ 항상 그렇다.

0 □ 전혀 아니거나 거의 드물다.

1 □ 가끔 그렇다.

2 □ 자주 그렇다.

3 □ 대부분 그렇다.

4 □ 항상 그렇다.

12. 1주일 동안 몇 번이나 대변을 봅니까?

*(가장 적합한 한가지만 고르세요)*

1 □ 1번 혹은 그 이하

2 □ 2

3 □ 3-4

4 □ 5-8

5 □ 9-12

6 □ 13-16

7 □ 17-21

8 □ 22-26

9 □ 27번 혹은 그 이상

13. 마지막으로 본 대변의 모양은?

*(가장 적합한 한가지만 고르세요)*


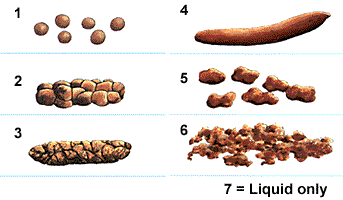


14. 최근 3개월 동안 대변이나 배변 후 변기에 피가 섞여 있었던 적이 있습니까? *(해당하는 것을* ***모두*** *고르세요)*

1 □ 단단해서 알갱이처럼 떨어졌다.

2 □ 단단하나 한 덩어리이다.

3 □ 소시지처럼 한 덩어리이나 표면이 갈라졌다.

4 □ 소시지처럼 한 덩어리이나 매끈하고 부드럽다.

5 □ 부드러운 여러 개의 덩어리이다.

6 □ 묽은 대변으로 변기에 흩어진다

7 □ 형태가 전혀 없이 물 같은 대변이다.

0 □ 아니요

1 □ 예– 선홍색 붉은 피

2 □ 예 – 자장 같은 검은색 대변

15. 최근 3개월 동안 단단하거나 덩어리지는 대변을 본적이 있습니까?

*(가장 적합한 한가지만 고르세요)*

16. 최근 3개월 동안 대변볼 때 과도하게 힘을 준 적이 있습니까?

*(가장 적합한 한가지만 고르세요)*

17. 최근 3개월 동안 대변을 본 후 대변이 항문에 남아 있는 것처럼 느낀 적(잔변감)이 있습니까?

0 □ 전혀 아니거나 거의 드물다.

1 □ 가끔 그렇다.

2 □ 자주 그렇다.

3 □ 대부분 그렇다.

4 □ 항상 그렇다.

0 □ 전혀 아니거나 거의 드물다.

1 □ 가끔 그렇다.

2 □ 자주 그렇다.

3 □ 대부분 그렇다.

4 □ 항상 그렇다.

0 □ 전혀 아니거나 거의 드물다.

1 □ 가끔 그렇다.

2 □ 자주 그렇다.

3 □ 대부분 그렇다.

4 □ 항상 그렇다.

18. 최근 3개월 동안 대변을 볼 때 대변이

잘 나가지 않고 막힌 것처럼 느낀 적이

있습니까?

*(가장 적합한 한가지만 고르세요)*

0 □ 전혀 아니거나 거의 드물다.

1 □ 가끔 그렇다.

2 □ 자주 그렇다.

3 □ 대부분 그렇다.

4 □ 항상 그렇다

19. 최근 3개월 간 대변을 보기 위해 항문 주위를 누르거나 손가락으로 대변을 제거한 적이 있습니까?

*(가장 적합한 한가지만 고르세요)*

20. 변비에 관한 위의 질문 15-19번 중 증상이 있다면, 그 증상은 6개월 전에 시작이 되었습니까?

21. 다음 중 변비로 인해 복용하거나 시행하고 있는 방법이 있습니까? (해당하는 것을 모두 고르세요)*.*

0 □ 전혀 아니거나 거의 드물다.

1 □ 가끔 그렇다.

2 □ 자주 그렇다.

3 □ 대부분 그렇다.

4 □ 항상 그렇다

0 □ 아니오

1 □ 예

0 □ 없다

1 □ 현미나 잡곡류

2 □ 식이섬유

3 □ 하제 (마그밀, 둘코락스, 아락실 등)

4 □ 관장

5 □ 좌약 (글리세린, 둘코락스등)

22. 최근 3개월 동안 묽은 변이나 물같이 풀어지는 대변을 본 적이 있습니까?

22A. 위의 증상이 6개월 전에 시작되었습니까?

0 □ 없거나 드물다. 질문 23으로 가세요

1 □ 가끔 그렇다.

2 □ 자주 그렇다.

3 □ 대부분 그렇다.

4 □ 항상 그렇다

0 □ 아니오

1 □ 예

22B. 위의 증상이 하제와 같은 변비약을 먹고 생겼습니까?

0 □ 아니오

1 □ 예

23. 최근 3개월 동안 변실금(화장실에 갈 때 까지 대변을 참지 못해 속옷을 묻히거나 누는 것)이 있었습니까?

0 □ 없음 질문 24으로 가세요

1 □ 1달에 한 번 미만

2 □ 1 달에 1일

3 □ 1달에 2-3 일

4 □ 1주일에 하루

5 □ 1주일에 2-6일

6 □ 매일

23A. 변실금이 있을 때 그 양은 어느정도입니까?

23B. 변실금이 있을 때 나오는 변의 형태는?

1 □ 속옷에 묻는 정도

2 □ 평소 배변하는 양보다는 적지만 속옷을 갈아입어야 할 정도

3 □ 평소 배변하는 정도로 겉옷까지 갈아입어야 할 정도

1 □ 물이나 점액만 나온다.

2 □ 대변만 나온다.

3 □ 물이나 점액과 대변이 같이 나온다.

| 다음은 다른 위장관 증상에 관한 질문입니다 |
| --- |

24. 최근 3개월 동안 명치부터 가슴쪽(그림 참조)으로 위로 뻗치는 것처럼 쓰리거나 타는 듯한 통증이 있었습니까? (단, 협심증이나 심장질환에 의한 통증은 제외하여 주십시오)

*(가장 적합한 한가지만 고르세요)*

24 A. 위의 증상이 6개월 전에 시작되었습니까?

24B. 최근 3개월 동안 입안으로 신물이 올라온 적이 있었습니까?

0 □ 없음 질문 25로 가세요.

1 □ 1달에 한 번 미만

2 □ 1 달에 1일

3 □ 1달에 2-3 일

4 □ 1주일에 하루

5 □ 1주일에 2-6일

6 □ 매일

0 □ 아니오

1 □ 예

가슴

•

•

0 □ 아니오

1 □ 예

25. 최근 3개월 동안 쉽게 배가 금방

불러와 식사를 조금밖에 못 먹은

적이 있었나요?

*(가장 적합한 한가지만 고르세요)*

26. 최근 3개월 동안 식사를 다 한 다음 윗배[명치]가 꽉 찬 느낌[내려가지 않는 느낌]이 있었나요?

*(가장 적합한 한가지만 고르세요)*

27. 위의 25-26 질문의 증상이 6개월 전에 시작되었습니까?

0 □ 없음

1 □ 1달에 한 번 미만

2 □ 1 달에 1일

3 □ 1달에 2-3 일

4 □ 1주일에 하루

5 □ 1주일에 2-6일

6 □ 매일

0 □ 없음

1 □ 1달에 한 번 미만

2 □ 1 달에 1일

•

•

3 □ 1달에 2-3 일

4 □ 1주일에 하루

5 □ 1주일에 2-6일

6 □ 매일

윗배

0 □ 아니오

1 □ 예

28. 최근 3개월 동안 윗배[명치]가

아프거나 쓰리 적이 있나요?

*(가장 적합한 한가지만 고르세요)*

28A. 상복부 통증 혹은 쓰린 증상은 어느 정도로 심했습니까?

28B. 이 증상은 6개월 이상 되었습니까?

0 □ 없음 질문 29로 가세요.

1 □ 1달에 한 번 미만

2 □ 1 달에 1일

3 □ 1달에 2-3 일

4 □ 1주일에 하루

5 □ 1주일에 2-6일

6 □ 매일

1 □ 아주 가벼워 대부분 잘 모른다.

2 □ 가벼운 정도로 생각하지 않으면 잘 모른다.

3 □ 어느 정도 있으나 일상생활에 영향을 주지 않는다.

4 □ 심해 일상생활에 영향을 준다.

5 □ 매우 심해 일상생활이 어렵다.

0 □ 아니오

1 □ 예

28C. 이러한 통증 혹은 쓰린 증상은 대변을 본 후 좋아집니까?

28D. 이러한 통증 혹은 쓰린

증상은 그 날로 없어 집니까?

0 □ 아니오

1 □ 예

0 □ 아니오

1 □ 예

| 28E. 이러한 통증 혹은 쓰린 증상은 0 □ 식사와 관계없다.  1 □ 식사를 하면 좋아진다  2 □ 식사를 하면 나빠진다 |
| --- |

29. 최근 3개월 동안, 복부 팽만감 혹은 복부 팽만이 있었습니까? *(가장 적합한 한가지만 고르세요)*

0 □ 없음 질문 30로 가세요.

1 □ 1달에 한 번 미만 2 □ 1 달에 1일

3 □ 1달에 2-3 일 4 □ 1주일에 하루

5 □ 1주일에 2-6일 6 □ 매일

29A. 복부팽만감이 있을 때, 실제로 배가 부어 오른 적이 있습니까?

0 □ 전혀 아니거나 거의 드물다.

1 □ 가끔 그렇다. 2 □ 자주 그렇다.

3 □ 대부분 그렇다. 4 □ 항상 그렇다.

29B. 이 증상은 6개월 이상 되었습니까?

0 □ 아니오 1 □ 예

30. 최근 3개월 동안, 구역질 혹은 메스꺼움이 있었습니까?

0 □ 없음

1 □ 1달에 한 번 미만 2 □ 1 달에 1일

3 □ 1달에 2-3 일 4 □ 1주일에 하루

5 □ 1주일에 2-6일 6 □ 매일

30A. 이 증상은 6개월 이상 되었습니까?

0 □ 아니오 1 □ 예

31. 최근 3개월 동안, 실제적으로 토한 적이 있습니까?

0 □ 없음

1 □ 1달에 한 번 미만 2 □ 1 달에 1일

3 □ 1달에 2-3 일 4 □ 1주일에 하루

5 □ 1주일에 2-6일 6 □ 매일

32. 최근 1년간 당신의 체중은?

0 □ 그대로다

1 □ 5kg 보다 적게 늘었다.

2 □ 5kg 이상 늘었다.

3 □ 5kg 보다 적게 줄었다.

4 □ 5kg 이상 줄었다.

33. 복부(배) 수술을 받은 적이 있습니까?

0 □ 없다***.***

1 □ 맹장 수술을 받았다.

2 □ 담낭절제술을 받았다.

3 □ 자궁 절제술을 받았다.

4 □ 기타________________________

34. 최근 1년간, 긴박뇨 (소변이 마려워 화장실에 급하게 가는 것) 가 있었습니까?

0 □ 전혀 아니거나 거의 드물다.

1 □ 가끔 그렇다.

2 □ 자주 그렇다.

3 □ 대부분 그렇다.

4 □ 항상 그렇다.

35. 최근 1년간, 요실금(화장실에 갈 때까지 소변을 참지못해 속옷에 묻히거나 누는 것)이 있었습니까?

0 □ 아니오

1 □ 예

- **여성인 경우만** 대답하여 주십시오.

36. 당신은 임신한 적이 있습니까? (단, 임신이 6개월 이상 지속된 경우만 포함하여 주십시오)?

0 □ 아니오

1 □ 예

임신이 6개월 이상 지속된 경우, 몇 번이나 출산하셨습니까? ________________

36A. (사산이 아닌) 정상출산은? ______________

36B. 제왕절개술은? _______________

36C. 정상 질식분만은? ____________

37. 현재 결혼상태는?

1 □ 미혼 혹은 독신

2 □ 결혼

3 □ 이혼

4 □ 배우자 사별

5 □ 기타

38. 현재 당신의 직업은?

1 □ 은퇴

2 □ 신체장애로 취업 불가능

3 □ 학생 혹은 주부

4 □ 무직 혹은 실직상태

5 □ 취직 상태

39. 당신의 최종학력은?

1 □ 무학

2 □ 초등학교 졸업

3 □ 중학교 졸업

4 □ 고등 학교 졸업

5 □ 전문대학교 졸업

6 □ 4년제 대학졸업

7 □ 대학원 이상

40. 당신의 키는? ( 센티미터)

*(센티미터로 답하여 주세요.)*

41. 당신의 몸무게는? ( 킬로그램)

*(킬로그램으로 답하여 주십시오; 예, 65 킬로그램)*

42. 당신은 규칙적으로 담배를 피십니까?

0 □ 아니오

1 □ 이전에 피웠으나 현재 금연했다

2 □ 예

43. 당신은 술을 얼마나 마십니까?

0 □ 아니거나 1달에 1회 이하

1 □ 1달에 2-3 회

2 □ 1주에 1-2회

3 □ 1 주에 3회 혹은 그 이상

4 □ 매일 마신다.

44. 당신의 아스피린(베이비 아스피린 포함)이나 진통 소염제를 드십니까?

1 □ 안 먹거나 한 달에 2-3회 이하

2 □ 1 주에 1-2회

3 □ 1주에 3-6회

4 □ 1주에 7-10회

5 □ 1주 10회 혹은 그 이상

45. 최근 10년간 의사에게 아래 질환이 있다고 들은 적이 있습니까?

45A. 각종 0 □ 아니오 1 □ 예

45B. 당뇨병 0 □ 아니오 1 □ 예

45C. 만성 신장 질환 0 □ 아니오 1 □ 예

46. 최근 1년간 당신은 의사를 몇 번 방문했습니까?

0 □ 없다.

1 □ 1-2 번

2 □ 3-5 번

3 □ 6-9번

4 □ 10회 이상

46A. 만약 의사를 방문한 적이 있다면, 특별히 위나 장 등 소화기 증상으로 방문한 적이 있습니까?

0 □ 아니오 1 □ 예

**최근 1년간 다음 증상이 1) 얼마나 자주 2) 얼마나 심한가를**

**양쪽 모두 표시하여 주세요 (없으면 “ 없음 “에 표시하여 주세요 )**

| **얼마나 자주 있나요?** | | | | | **양쪽** 모두 표시 하여 주세요 | **얼마나 심하신가요?** | | | | |
| --- | --- | --- | --- | --- | --- | --- | --- | --- | --- | --- |
| 없음 | 1달  한번  이상 | 1주  한번  이상 | 1주  여러 번 | 매일 |  | 없음 | 약간  불편 | 꽤  불편 | 심함 | 매우  심함 |
|  |  |  |  |  | 1. 두통 |  |  |  |  |  |
|  |  |  |  |  | 2. 등이나 허리통증 |  |  |  |  |  |
|  |  |  |  |  | 3. 천식[쌕쌕거린다] |  |  |  |  |  |
|  |  |  |  |  | 4. 숨이 차다 |  |  |  |  |  |
|  |  |  |  |  | 5. 불면증[잠자기  어렵다] |  |  |  |  |  |
|  |  |  |  |  | 6. 피로감 |  |  |  |  |  |
|  |  |  |  |  | 7. 온 몸이 뻣뻣하다 |  |  |  |  |  |
|  |  |  |  |  | 8. 심장이 두근거리  거나 뛴다 |  |  |  |  |  |
|  |  |  |  |  | 9. 관절통 |  |  |  |  |  |
|  |  |  |  |  | 10. 우울하다 |  |  |  |  |  |
|  |  |  |  |  | 11. 글자를 볼 때  눈이 아프다 |  |  |  |  |  |
|  |  |  |  |  | 12.어지럽다[현기증] |  |  |  |  |  |
|  |  |  |  |  | 13. 전신 쇠약감  [힘이 없다] |  |  |  |  |  |
|  |  |  |  |  | 14. 신경이 과민하거나쉽게 불안해진다 |  |  |  |  |  |
|  |  |  |  |  | 15. 갑자가 덥거나  갑자기 추워진다 |  |  |  |  |  |
|  |  |  |  |  | 16. 혈압이 높다 |  |  |  |  |  |
|  |  |  |  |  | 17. 걱정스럽고  두려운 느낌이 든다 |  |  |  |  |  |

**빠짐없이 표시하셨는지 다시 한 번 보아 주십시오. 감사합니다.**

다음의 문항들은 **어려운 문제나 힘든 일이 생겼을 때,** 여러분이 어떻게 하는가를 알아보기 위한 것입니다. 지난 3개월 동안 가장 힘들었던 일은 무엇입니까? 그런 일이 있었을 때 어떻게 하셨는지 잘 생각해 보시고 **다음 문항에 숫자로** 답해 주십시오.

0 1 2 3

전혀 그렇지 그렇치 않은 조금 그렇다 매우 그렇다

않다 편이다.

1. 문제를 해결하기 위해 여러 가지 방법을 생각해 보았다. ( )
2. 상황에 대해 이모저모 더 알아보았다 ( )
3. 문제에 대해 배우자나 친척들과 얘기해 보았다 ( )
4. 긍정적인 측면을 보도록 노력하였다. ( )
5. 도움이나 힘을 얻을수 있도록 기도하였다 ( )
6. 문제에 대해 친구와 얘기하였다 ( )
7. 전문가(의사, 법률가)와 얘기해 보았다. ( )
8. 그 일을 떨쳐버리려고 다른 일들을 바쁘게 하였다. ( )
9. 무엇을 할것인지 계획을 세우고 그대로 해 보았다 ( )
10. 화나거나 우울할 때 다른 사람에게 짜증을 내었다 ( )
11. 감정을 마음속에 쌓아두었다 ( )
12. 평소보다 더 많이 먹었다 ( )
13. 과거에 이와 비슷한 일이 있었을 때 어떻게 했는지 곰곰히 생각해 보았다( )

0 1 2 3

전혀 그렇지 그렇치 않은 조금 그렇다 매우 그렇다

않다 편이다.

1. 한번에 한가지씩 차근차근 생각해 보았다. ( )
2. 시장이나 백화점을 돌아다니면서 구경하거나 물건을 샀다 ( )
3. 평소보다 술을 더 마셨다 ( )
4. 사람들과 있는 것을 피했다 ( )
5. 그러한 일이 일어났다는 것을 믿지 않으려 하였다 ( )
6. 평소보다 담배를 더 많이 피웠다 ( )
7. 평소보다 잠을 더 많이 잤다 ( )
8. 해야할 일을 더 노력해서 실행에 옮겼다 ( )
9. 어떻게든지 내 기문을 표현해 보았다 ( )
10. 기분이 좋아질 만한 일들을 떠올려 보았다 ( )
11. 비슷한 경험들을 가진 사람들로부터 도움을 구해 보았다 ( )
12. 힘든 상황이지만 그 속에서 뭔가를 조금이라도 얻어내려고 노력했다 ( )
13. 다음 번에는 달라질수 있도록 스스로에게 다짐하였다. ( )
14. 문제는 있는 그대로 받아들였다 ( )
15. 신경안정제를 복용하였다 ( )
16. 운동을 더 열심히 해서 긴장을 풀도록 노력하였다 ( )

**담낭절제 후 위장증상 조사 (Phase 2)**

**응답자 ID ___________________________________**

**** 위장관 증상에 관한 질문 ****

**A1.** 최근 3개월 동안 배가 아프거나 불편한 적이 있었나요?

0. 없음 →[A7로 가시오] 1. 있음 →[A1-1로 가시오]

A1-1. 배가 아프거나 불편한 증상은 언제 시작 되었습니까?

1. 6개월 이전 2. 6개월 이후

A1-2. 배가 아프거나 불편한 증상은 얼마나 자주 있나요?

1. 한 달에 한 번 미만 2. 한 달에 1일

3. 한 달에 2-3번 4. 1주일에 1일

5. 1주일에 2-6일 6. 매일

***** 최근 3개월 동안 경험하신 배가 아프거나 불편한 경험[A1]과 관련하여 다음 불러드리는 각

문장들에 대해 본인의 상황을 잘 설명하는 보기 한 가지만 골라주세요

|  | **전혀 아니거나**  **거의 드물다** | **가끔**  **그렇다** | **자주**  **그렇다** | **대부분**  **그렇다** | **항상**  **그렇다** |
| --- | --- | --- | --- | --- | --- |
| **A2.** 복부 통증이나 불편한 증상이 대변을 보고 나면 좋아지나요? | 1 | 2 | 3 | 4 | 5 |
| **A3.** 복부 통증이나 불편한 증상이 있을 때 대변을 더 자주 보나요? | 1 | 2 | 3 | 4 | 5 |
| **A4.** 복부 통증이나 불편한 증상이 있을 때 대변을 더 드물게 보나요? | 1 | 2 | 3 | 4 | 5 |
| **A5.** 복부 통증이나 불편한 증상이 있을 때 대변이 무르거나 풀어지나요? | 1 | 2 | 3 | 4 | 5 |
| **A6.** 복부 통증이나 불편한 증상이 있을 때 대변이 더 단단하거나 굳어지나요? | 1 | 2 | 3 | 4 | 5 |

* 다음 각 질문에 대해 본인의 상황을 잘 설명하는 보기 한 가지만 골라주세요

윗배 또는 명치란 가슴뼈 아래 중앙의 오목하게 들어간 부분을 말합니다.

| **해당하는 답을 하나만 골라 Ⅴ표시 해 주십시오** | **없음** | **1달에**  **한번 미만** | **1달에**  **1일** | **1달에**  **2-3일** | **1주일에 하루** | **1주일에**  **2-6일** | **매일** | |
| --- | --- | --- | --- | --- | --- | --- | --- | --- |
| **A7.** 최근 3개월 동안 가슴이 쓰리거나 화끈 거리거나 타는 듯한 적이 있었나요?l | 0 | 1 | 2 | 3 | 4 | 5 | 6 |  |
| A7-1. 6개월 전에 시작되었습니까? | 0 □ 아니오 1 □ 예 | | | | | | |  |
| **A8.** 최근 3개월 동안 식사 도중 배가 불러와 보통 먹는 식사량을 다 먹지 못한 적이 있었습니까? | 0 | 1 | 2 | 3 | 4 | 5 | 6 |  |
| A8-1. 6개월 전에 시작되었습니까? | 0 □ 아니오 1 □ 예 | | | | | | |  |
| **A9.** 최근 3개월 동안 보통 먹는 식사량을 다 먹은 후 윗배[명치]가 꽉 찬 느낌[내려가지 않는 느낌]이 있었습니까? | 0 | 1 | 2 | 3 | 4 | 5 | 6 |  |
| A9-1. 6개월 전에 시작되었습니까? | 0 □ 아니오 1 □ 예 | | | | | | |  |
| **A10.** 최근 3개월 동안 윗배[명치]에 통증이나 쓰린 증상이 있었습니까? | 0 | 1 | 2 | 3 | 4 | 5 | 6 |  |
| A10-1. 윗배[명치]에 통증이나 쓰린 증상이 6개월 전에 시작되었나요? 0. 아니오 1. 예 | | | | | | | |  |
| A10-2. 윗배[명치]에 통증이나 쓰린 증상이 어느 정도로 심한가요?  1. 매우 약함 2. 약함 3. 보통 4. 심함 5. 매우 심함 | | | | | | | |  |

| **해당하는 답을 하나만 골라 Ⅴ표시 해 주십시오** | | **없음** | **1달에**  **한 번미만** | **1달에**  **1일** | **1달에**  **2-3일** | **1주일에**  **1일** | **1주일에2-6일** | **매일** |
| --- | --- | --- | --- | --- | --- | --- | --- | --- |
| **A11.** | 최근 3개월 동안 신물이 올라오는 증상이 있었나요 | 0 | 1 | 2 | 3 | 4 | 5 | 6 |
| **A12.** | 최근 3개월 동안 구역감  [속이 울렁거리는 증상]이 있었나요? | 0 | 1 | 2 | 3 | 4 | 5 | 6 |
| **A13.** | 최근 3개월 동안 토한적이 있었나요? | 0 | 1 | 2 | 3 | 4 | 5 | 6 |
| **A14.** | 최근 3개월 동안 배가 불러오거나 빵빵한 느낌이 있었나요? | 0 | 1 | 2 | 3 | 4 | 5 | 6 |

**** 용변 관련 질문 ****

| **해당하는 답을 하나만 골라 √ 표시 해**  **주십시오.** | **전혀 아니거나 거의 드물다** | **가끔**  **그렇다** | **자주**  **그렇다** | **대부분 그렇다** | **항상**  **그렇다** |
| --- | --- | --- | --- | --- | --- |
| **A24.** 최근 3개월 동안 단단하거나 덩어리지는 대변을 본적이 있습니까? | 1 | 2 | 3 | 4 | 5 |
| **A25.** 최근 3개월 동안 대변볼 때 과도하게 힘을 준 적이 있습니까? | 1 | 2 | 3 | 4 | 5 |
| **A26.** 최근 3개월 동안 대변을 본 후 대변이 항문에 남아 있는 것처럼 느낀 적[잔변감]이 있습니까? | 1 | 2 | 3 | 4 | 5 |
| **A27.** 최근 3개월 동안 대변을 볼 때 대변이 잘 나가지 않고 막힌 것처럼 느낀 적이 있습니까? | 1 | 2 | 3 | 4 | 5 |
| **A28.** 최근 3개월 동안 대변을 보기 위해 항문 주위를 누르거나 손가락으로 대변을 제거한 적이 있습니까? | 1 | 2 | 3 | 4 | 5 |
| **A29.** 최근 3개월 동안 묽은 변이나 물같이 풀어지는 대변을 본 적이 있습니까? | 1 | 2 | 3 | 4 | 5 |

**A30.** 당신은 1주일 동안 몇 번이나 대변을 보십니까? ____________________________회

감사합니다.

# Supplementary Figures and Tables

## Supplementary Figure 1. Prevalence of functional gastrointestinal disorders in phase 1 and phase 2. FGID, functional gastrointestinal disorder; IBS, irritable bowel syndrome; Ch. abdominal pain, chronic abdominal pain.

2.2 Supplementary Tables

Supplementary Table 1. Univariate and multivariate analysis of predictors for new-onset functional dyspepsia one year after cholecystectomy

|  | Univariate | | | Multivariate | | |
| --- | --- | --- | --- | --- | --- | --- |
|  | OR | 95% CI | *P*  value | OR | 95% CI | *P* value |
| Male gender | 0.503 | 0.2269-0.940 | 0.031 | 0.644 | 0.331-1.252 | 0.194 |
| Age (years) |  |  |  |  |  |  |
| ≤ 30 | 1.300 | 0.454-3.716 | 0.625 | 1.453 | 0.486-4.346 | 0.504 |
| 31-50 | 0.848 | 0.453-1.588 | 0.607 | 1.058 | 0.540-2.072 | 0.870 |
| ≥ 51 | 1 | - | 1 | 1 | - | 1 |
| BMI | 1.001 | 0.894-1.121 | 0.986 | - | - | - |
| DM | 0.435 | 0.058-3.267 | 0.418 | - | - | - |
| Cholecystectomy | 2.322 | 1.069-5.042 | 0.033 | 2.294 | 1.023-5.147 | 0.044 |
| SSC score | 1.885 | 1.482-2.397 | < 0.001 | 1.815 | 1.426-2.309 | < 0.001 |

OR, odd ratio; CI, confidence interval; BMI, body mass index; DM, diabetes mellitus; SSC, somatic symptom checklist

Supplementary Table 2. Univariate and multivariate analysis of predictors for new-onset chronic abdominal pain one year after cholecystectomy

|  | Univariate | | | Multivariate | | |
| --- | --- | --- | --- | --- | --- | --- |
|  | OR | 95% CI | *P* value | OR | 95% CI | *P* value |
| Male gender | 0.922 | 0.450-1.888 | 0.824 | 1.175 | 0.548-2.518 | 0.679 |
| Age (years) |  |  |  |  |  |  |
| ≤ 30 | 0.538 | 0.066-4.373 | 0.562 | 0.662 | 0.079-5.573 | 0.704 |
| 31-50 | 1.276 | 0.576-2.827 | 0.548 | 1.744 | 0.732-4.153 | 0.209 |
| ≥ 51 | 1 | 1 | 1 | 1 | - | 1 |
| BMI | 0.948 | 0.832-1.080 | 0.420 | - | - | - |
| DM | 1.290 | 0.294-5.661 | 0.736 | - | - | - |
| Cholecystectomy | 2.897 | 1.195-7.020 | 0.019 | 2.618 | 1.050-6.527 | 0.039 |
| SSC score | 1.870 | 1.375-2.544 | < 0.001 | 1.941 | 1.407-2.678 | < 0.001 |

OR, odd ratio; CI, confidence interval; BMI, body mass index; DM, diabetes mellitus; SSC, somatic symptom checklist

Supplementary Table 3. Univariate and multivariate analysis of predictors for new-onset chronic diarrhea one year after cholecystectomy

|  | Univariate | | | Multivariate | | |
| --- | --- | --- | --- | --- | --- | --- |
|  | OR | 95% CI | *P* value | OR | 95% CI | *P* value |
| Male gender | 1.244 | 0.25-6.20 | 0.790 | 1.44 | 0.26-7.96 | 0.676 |
| Age (years) |  |  |  |  |  |  |
| ≤ 30 | - | - | 0.995 | - | - | 0.994 |
| 31-50 | - | - | 0.995 | - | - | 0.995 |
| ≥ 51 | 1 | - | 1 | 1 | - | 1 |
| BMI | 1.223 | 0.98 -1.53 | 0.079 | - | - | - |
| DM | 0 | - | 0.998 | - | - | - |
| Cholecystectomy | 44.93 | 5.18- 389.97 | 0.001 | 40.03 | 4.50-356.26 | 0.001 |
| SSC score | 1.43 | 0.98 - 2.09 | 0.063 | 1.309 | 0.767-2.236 | 0.324 |

OR, odd ratio; CI, confidence interval; BMI, body mass index; DM, diabetes mellitus; SSC, somatic symptom checklist
