# Supplementary figures and images for: Development of functional gastrointestinal disorder symptoms following laparoscopic cholecystectomy: a prospective cohort study
Source: Front Med (Lausanne). 2023 Oct 6;10:1248465. doi: 10.3389/fmed.2023.1248465 (PMC10587431; doi:10.3389/fmed.2023.1248465)

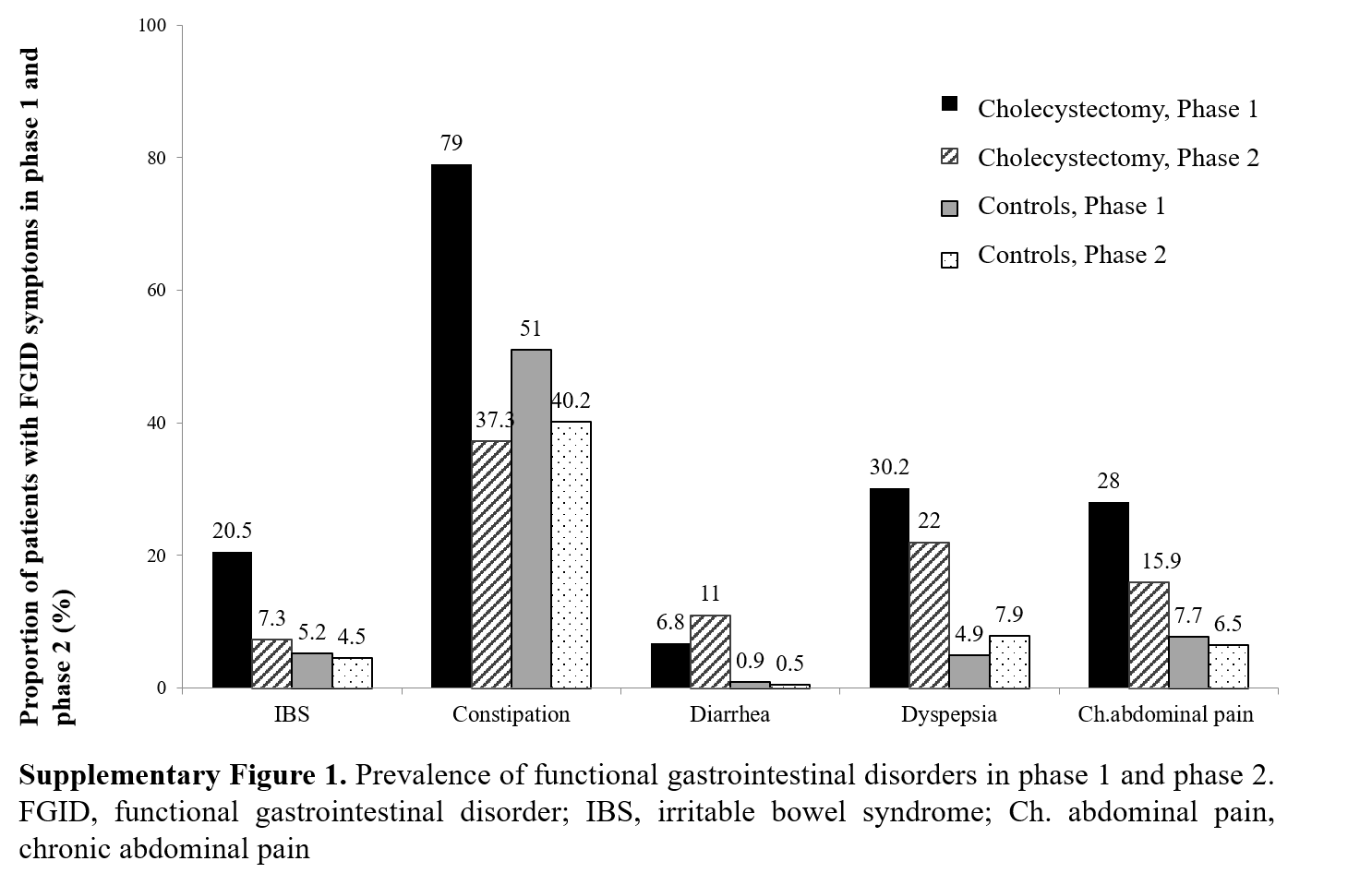

Supplement: Supplementary file 2 [file Image_1.TIF]
